# Inherited factors contribute to an inverse association between preeclampsia and breast cancer

**DOI:** 10.1186/s13058-017-0930-6

**Published:** 2018-01-23

**Authors:** Haomin Yang, Wei He, Mikael Eriksson, Jingmei Li, Natalie Holowko, Flaminia Chiesa, Per Hall, Kamila Czene

**Affiliations:** 1 Department of Medical Epidemiology and Biostatistics, Karolinska Institutet, SE-17177 Stockholm, Sweden; 2 Genome Institute of Singapore, 138672 Singapore, Singapore; 3 Department of Oncology, South General Hospital, SE-11883 Stockholm, Sweden

**Keywords:** Breast cancer, Preeclampsia, Mammographic density

## Abstract

**Background:**

Preeclampsia is frequently linked to reduced breast cancer risk. However, little is known regarding the underlying genetic association and the association between preeclampsia and mammographic density.

**Methods:**

This study estimates the incidence rate ratios (IRRs) of breast cancer in patients with preeclampsia, when compared to women without preeclampsia, using Poisson regression models in two cohorts of pregnant women: a Swedish nationwide cohort (n = 1,337,934, 1973–2011) and the Karolinska Mammography Project for Risk Prediction of Breast Cancer (KARMA, n = 55,044, 1958–2015). To identify the genetic association between preeclampsia and breast cancer, we used logistic regression models to calculate the odds ratios (ORs) of preeclampsia in sisters of breast cancer patients, and in women with different percentiles of breast cancer polygenic risk scores (PRS). Linear regression models were used to estimate the mammographic density by preeclampsia status in the KARMA cohort.

**Results:**

A decreased risk of breast cancer was observed among patients with preeclampsia in both the nationwide (IRR = 0.90, 95% CI = 0.85; 0.96) and KARMA cohorts (IRR = 0.75, 95% CI = 0.61; 0.93). Women with high breast cancer PRS and sisters of breast cancer patients had a lower risk of preeclampsia (OR = 0.89, 95% CI = 0.83; 0.96). Mammographic density was lower in women with preeclampsia compared to women without preeclampsia (-2.04%, 95% CI = -2.65; -1.43). Additionally, among sisters in the KARMA cohort (N = 3500), density was lower in sisters of patients with preeclampsia compared to sisters of women without preeclampsia (-2.76%, 95% CI = -4.96; -0.56).

**Conclusion:**

Preeclampsia is associated with reduced risk of breast cancer and mammographic density. Inherited factors contribute to this inverse association.

**Electronic supplementary material:**

The online version of this article (doi:10.1186/s13058-017-0930-6) contains supplementary material, which is available to authorized users.

## Background

Preeclampsia is a pregnancy-related disease originating from the placenta and characterized by hypertension and proteinuria [1]. Preeclampsia occurs in 3–5% of pregnancies and can cause life threatening complications, including stroke, eclampsia, placental abruption and renal failure [2]. While preeclampsia is associated with a long-term increased risk of cardiovascular disease and overall mortality, it is generally not associated with increased risk of cancer [3].

While an inverse association between preeclampsia and breast cancer risk has consistently been shown since the 1980s [4–7], several studies have reported conflicting results. This may be due to limitations in the number of breast cancer diagnoses after preeclampsia, the use of case-control study design or genetic heterogeneity of the studied populations [8–10]. Previous epidemiological studies investigating the association between breast cancer and preeclampsia by different reproductive characteristics of the women have yielded less conclusive results [4, 6]. The contradictory results from populations of European and Asian ancestry suggest that genetic components might influence the association between these two diseases [10]. Despite this, evidence for a genetic association between preeclampsia and breast cancer is scarce [11].

Although it is hypothesized that hormonal changes due to preeclampsia are associated with mammary gland development, and a subsequent reduction in breast cancer risk [12], we are unaware of any studies evaluating the association between preeclampsia and mammographic density. Mammographic density refers to the percentage of radiologically dense fibro-tissue identified through breast imaging, and is widely considered to be an intermediate phenotype for breast cancer [13, 14]. It can therefore be used as a powerful proxy when investigating the association between preeclampsia and breast cancer.

This study assessed the risk of breast cancer after preeclampsia diagnosis, using Swedish population-based registers. We further investigated the genetic association between preeclampsia and breast cancer, by testing the risk of preeclampsia in cancer-free sisters of patients with breast cancer and in women with different polygenic risk scores (PRS) for breast cancer. The association between preeclampsia and mammographic density was also analyzed in the mammographic screening cohort to confirm this biological association.

## Methods

### Study populations

**Fig. 1:**
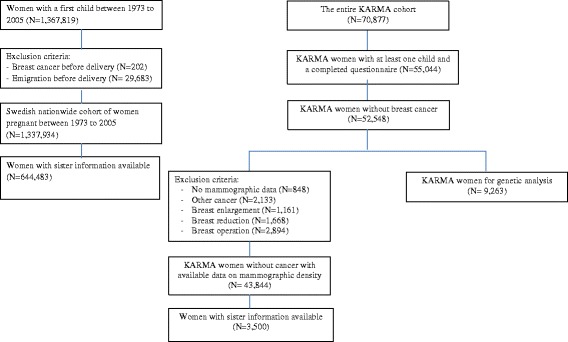
Flow chart of study population

This study included two cohorts: (1) a Swedish nationwide cohort of pregnant women (nationwide cohort in short) and (2) a Swedish mammographic screening-based cohort (Karolinska Mammography project for risk prediction of breast cancer, KARMA) (Fig. 1).

Data on the nationwide cohort was retrieved from the Swedish Medical Birth Register, which contains data on more than 99% of all births [15], and includes all women who delivered their first child between 1973 and 2005 (N = 1,337,934). Pregnancy characteristics including height, pre-pregnancy weight, smoking status, and previous reproductive history were collected at the first antenatal visit (at approximately 8–12 weeks of gestation). Education level was collected from the Swedish Register of Education. Information on sisters of the women was obtained by linking the cohort to the Swedish Multi-Generation Register. Maternal age and diseases related to pregnancy are reported by clinicians on post-delivery hospital discharge. These diseases are registered according to the Swedish version of the International Classification of Diseases (ICD), and for preeclampsia is coded as follows; ICD-10 (1997 to the present): O14 and O15; ICD-9 (1987-1996): 642E, 642F and 642G; and ICD-8 (1969-1986): 63703, 63704, 63709, 63710 and 63799.

The KARMA cohort included 70,877 women attending mammography screening or clinical mammography at one of four hospitals in Sweden between 2011 and 2013 [16]. Apart from mammographic imaging and blood sample collection, the participants answered a web-based questionnaire covering demographic, anthropometric, reproductive, and lifestyle risk factors related to breast cancer (selected basic information in Table 1). Information about preeclampsia diagnosis was also sought in the questionnaire. We linked the KARMA cohort to the Swedish Multi-Generation Register to obtain information on sister relationships and subsequently identified sisters of the patients with preeclampsia. For this study, we only included women who delivered their first child after 1958 (considering the start of the cancer register), and completed the full questionnaire (N = 55,044).

**Table 1:** Subject characteristics of the nationwide cohort and KARMA cohort

	No. of women (%)	
Variable names	Non-PE	PE patients
Nationwide cohort		
Number of women	1,270,353	67,581
Age at first birth		
<25	518,407 (40.8)	27,733 (41.0)
25-30	467,368 (36.8)	23,967 (35.5)
30-35	216,920 (17.1)	11,638 (17.2)
>35	67,658 (5.33)	4243 (6.28)
Number of births		
1	348,710 (27.5)	16,650 (24.6)
2	614,068 (48.3)	32,128 (47.5)
>=3	307,575 (24.2)	18,803 (27.8)
Weight status (by BMI)		
Underweight (<18.5)	39,033 (4.21)	1136 (2.18)
Healthy weight (18.5-24.9)	636,349 (68.7)	27,641 (53.1)
Overweight (25.0-29.9)	186,711 (20.2)	14,461 (27.8)
Obese ( ≥30.0)	64,726 (6.98)	8775 (16.9)
Smoking status (cigarettes/day)		
No	670,294 (80.1)	40,015 (85.6)
1-9 cigarettes	112,104 (13.4)	4675 (10.0)
>9 cigarettes	54,543 (6.52)	2068 (4.42)
Education Level		
Elementary	135,442 (10.7)	7000 (10.4)
Intermediate	615,696 (48.5)	34,978 (51.8)
College	499,481 (39.3)	24,939 (36.9)
Other	19,734 (1.55)	664 (0.98)
KARMA cohort		
Number of women	52,222	2822
Mean age at mammography (SD)	55.3 (9.9)	53.2 (9.2)
Average percent mammographic density (SD)	22.3 (19.5)	19.3 (18.9)
Menopausal status at mammography		
Pre-menopausal	20,774 (39.8)	1335 (47.3)
Peri-menopausal	1779 (3.41)	138 (4.89)
Post-menopausal	29,669 (56.8)	1349 (47.8)
Age at first birth		
<25	17,666 (33.8)	938 (33.2)
25-30	18,594 (35.6)	942 (33.4)
30-35	11,072 (21.2)	606 (21.5)
>35	4890 (9.36)	336 (11.9)
Number of births		
1	8843 (16.9)	448 (15.9)
2	28,741 (55.0)	1469 (52.1)
>=3	14,638 (28.0)	905 (32.1)
Weight status (by BMI)		
Underweight (<18.5)	486 (0.93)	22 (0.78)
Healthy weight (18.5-24.9)	28,860 (55.3)	1194 (42.3)
Overweight (25.0-29.9)	16,482 (31.6)	1011 (35.8)
Obese ( ≥30.0)	6215 (11.9)	583 (20.7)
Smoking status (cigarettes/day)		
No	5172 (9.90)	302 (10.7)
1-9 cigarettes	13,425 (25.7)	625 (22.2)
>9 cigarettes	8926 (17.1)	445 (15.8)
Education Level		
Elementary	3933 (7.55)	171 (6.07)
Intermediate	16,176 (31.1)	998 (35.4)
College	26,861 (51.6)	1465 (52.0)
Other	5105 (9.80)	184 (6.53)
Alcohol use		
Never	9566 (18.5)	700 (24.9)
0-5g/day	13,666 (26.4)	697 (24.8)
5-10g/day	18,553 (35.8)	980 (34.9)
>10g/day	10,071 (19.4)	435 (15.5)
Age at menarche		
<12	17,325 (33.9)	1133 (41.0)
13-16	31,272 (61.1)	1531 (55.4)
>16	2576 (5.03)	102 (3.69)
Body shape at age 18*		
1	2328 (4.46)	130 (4.61)
2	12,644 (24.2)	557 (19.7)
3	19,074 (36.5)	952 (33.7)
4	12,990 (24.9)	805 (28.5)
5~9	5025 (9.62)	374 (13.3)
Hours of Physical activity at age 18 (per week)		
<1	8356 (17.4)	458 (17.7)
1-2	14,193 (29.5)	725 (28.0)
3-5	14,082 (29.3)	776 (30.0)
≥5	11,459 (23.8)	628 (24.3)
Irregular menstrual cycle in adult life		
No	45,465 (87.1)	2382 (84.4)
Yes	5799 (11.1)	391 (13.9)

Abbreviations: No.=Number; PE=Preeclampsia; BMI= Body mass index. The nationwide cohort includes Swedish women who delivered their first child between 1973 and 2005. In this cohort, follow-up is complete until December 31, 2011. The KARMA cohort includes women who participated in mammographic screening or clinical mammography program between 2011 and 2013, and all women in this cohort have complete follow-up until Feb 28, 2015. * Body shape was defined according to the Stunkard Figure Rating Scale in the supplemental figure 1

Follow up of the nationwide cohort started from the date of birth of the first child (see above), and ended on the date of first breast cancer diagnosis, date of death, date of emigration or end of follow up (31 December 2011), whichever came first. Information on breast cancer diagnosis, death and emigration was obtained through cross-linking the cohort to the Swedish Cancer Register, Swedish Causes of Death Register and the Swedish Migration Register, using unique Swedish personal identification numbers [17]. Breast cancer diagnosis was based on ICD-7 code 170 in the cancer register. The Swedish Cancer register started from 1958 and is considered to have almost 100% completeness [18, 19]. Follow up of the KARMA cohort also started from the date of birth of the first child, and ended the same time as the nationwide cohort, except for an extension of the follow up until 28 February 2015. The study was approved by the Regional Ethical Review Board in Stockholm, Sweden.

### Mammographic density measurement

Mammograms in the mediolateral oblique (MLO) position were obtained from four Swedish hospitals in women participating in the KARMA cohort during 2011–2013. The fully automated software STRATUS was used to measure area-based mammographic density (details of this method have been described elsewhere) [20]. STRATUS measures mammographic density regardless of vendor of mammography machine and thus ensures comparability of mammographic density at the population level. The percentage density was calculated by dividing the dense area by the total breast area in the mammogram. Women were excluded from the mammographic density analysis if they had any previous cancers, or any breast enlargement or reducing surgery, leaving 43,844 women available for this analysis.

### Polygenic risk score

Blood samples from a subset of 9263 women without breast cancer from the KARMA cohort were genotyped using a custom Illumina iSelect array (iCOGS), comprising 211,155 single nucleotide polymorphisms (SNPs) [21], or an Illumina Infinium OncoArray, comprising 499,170 SNPs [22]. Details of the array design, sample handling and quality control processes are described elsewhere [21, 22]. To assess genetic predisposition to breast cancer, we selected 171 genome-wide significant SNPs reported in a recent meta-analysis of breast cancer genome-wide association studies (GWAS) for constructing a PRS [22]. These SNPs were imputed using the 1000 Genomes Project March 2012 release as a reference [23] and passed quality control. For each individual, a weighted PRS was calculated using the following formula:

$$ PRS={\beta}_1{x}_1+{\beta}_2{x}_2+.\dots {\beta}_k{x}_k+{\beta}_n{x}_n $$

where *β* is the per-allele log odds ratio (OR) of breast cancer associated risk allele for SNP _*k*_*, x*_*k*_ is the number of alleles for the same SNP (0, 1, 2), and _*n*_ is the total number of the disease SNPs included in the profile. The SNPs and corresponding log ORs (weights) used for the derivation of PRS are summarized in Additional file 1: Table S1. For analysis, women were categorized in the following percentiles of breast cancer risk based on PRS: 0–40%, 40–60%, 60–80%, 80–90% and 90–100%.

### Statistical analysis

An age-adjusted incidence rate of breast cancer was calculated in both the nationwide and KARMA cohorts, taking the 1990 Swedish national census population as the standard population. Considering the few cases of breast cancer in the age category 70–80 years in the KARMA cohort (n = 5), the age-adjusted incidence rate was restricted to an age band of 20–70 years. Poisson regression models were used to calculate incidence rate ratios for breast cancer in patients with preeclampsia. In this analysis, preeclampsia was considered as a time varying exposure, in which the exposed person-time was counted from the time of preeclampsia diagnosis. The underlying time scale was attained age. We constructed two models to analyze the association between preeclampsia and breast cancer incidence: (1) a basic model (model 1) adjusted for calendar period (10-year categories) and (2) model 2: with additional adjustment for number of births (time varying covariate), age at first birth, weight status categories (based on World Health Organization (WHO) body mass index (BMI) cutoff points (underweight (<18.5), healthy weight (18.5–24.9), overweight (25.0–29.9), obese (≥ 30.0)), smoking status and education level. In the analysis of the KARMA cohort, model 2 was additionally adjusted for alcohol use, age at menarche, physical activity at age 18 years, body shape at age 18 years (detailed information on body shape categories has been described elsewhere and shown in Additional file 1: Figure S1) [24], and irregular menstrual cycles in adult life. We also conducted two additional analyses to further adjust for breast cancer PRS on the basis of model 2, and to separately evaluate the risk of estrogen receptor positive (ER+) and negative (ER-) breast cancer in the KARMA cohort.

To identify the genetic association between preeclampsia and breast cancer, we used logistic regression models to estimate the ORs of preeclampsia (as an outcome) among cancer-free sisters of the patients with breast cancer, compared to women in the nationwide cohort without history of breast cancer and without a sister with history of breast cancer, adjusting for number of births. We also calculated the OR of preeclampsia by percentiles of breast cancer PRS for women in the KARMA cohort who did not have breast cancer and were genotyped, adjusting for number of births and batch effect of genotyping. For both of the analyses, we additionally adjusted for age at first birth, weight status categories, smoking status and education level in model 2.

As mammographic density is widely considered to be an intermediate phenotype of breast cancer, we tested the association between percentage mammographic density and previous diagnosis of preeclampsia in cancer-free women in KARMA. For this analysis, linear regression models with robust “sandwich” standard errors for confidence intervals were used, to avoid assuming normally distributed error terms and homoscedastic variance of the outcomes. We only adjusted for age at mammogram (continuous) in model 1, and additionally for BMI categories, age at menarche, number of births, age at first birth, menopausal status at mammogram, irregular menstrual cycle, physical activity at age 18 years, body shape at age 18 years, education level, smoking status and alcohol consumption in model 2. In order to test the genetic association between preeclampsia and mammographic density, we selected the cancer-free women in KARMA who have a sister in the cohort (N = 3500) and investigated the differences of mammographic density in sisters of patients with preeclampsia, compared to women without a sister with preeclampsia.

Statistical analyses were performed using SAS (version 9.4; SAS Institute Inc, Cary, NC, USA) and Stata software (version 14.0; Stata Corporation, College Station, TX, USA), at a two-tailed alpha level of 0.05.

## Results

Table 1 shows subject characteristics of women in the nationwide cohort and the KARMA cohort. In both cohorts, approximately 5–6% of women had preeclampsia. Preeclampsia was more frequently observed in women with an older age at first birth, higher parity, higher BMI, less cigarette smoking and higher education level.

### Preeclampsia and subsequent risk of breast cancer

In the Swedish nationwide cohort of pregnant women, 27,626 of the 1,337,934 women developed breast cancer during a median follow up of 21.6 years, corresponding to an age-adjusted incidence rate of 1.5/1000 person years (20–70 years old). Compared to women without history of preeclampsia, patients with preeclampsia had 10% decreased risk of breast cancer (IRR = 0.90, 95% CI = 0.85; 0.96) in the multivariable adjusted model. Furthermore, the reduced risk of breast cancer was even lower in women with repeated occurrence (two or more times) of preeclampsia (IRR = 0.81, 95% CI = 0.66; 0.99) (Table 2). In the KARMA cohort, 2496 of the 55,044 women developed breast cancer during a median of 29.2 years of follow up, corresponding to an age-adjusted incidence rate of 3.0/1000 person years (20–70 years old) and a 25% decreased risk of breast cancer in women with preeclampsia (IRR = 0.75, 95% CI = 0.61; 0.93). A further adjustment for breast cancer PRS slightly attenuated the IRR to 0.83 (95% CI = 0.65; 1.06). The IRRs for ER+ and ER- breast cancer were 0.80 (95% CI = 0.61; 1.05) and 0.76 (95% CI = 0.36; 1.62), suggesting the inverse association between preeclampsia and breast cancer did not differ much according to cancer ER status.

**Table 2:** Association between preeclampsia and breast cancer among the nationwide cohort and KARMA cohort

Condition	Number of breast cancer cases	IRR (95% CI)	
		Model 1	Model 2
Nationwide cohort (*N* = 1,337,934)			
Preeclampsia			
No	26447	1.00 (REF)	1.00 (REF)
Yes	1179	**0.88 (0.83; 0.94)**	**0.90 (0.85; 0.96)**
once	1082	**0.90 (0.84; 0.95)**	**0.91 (0.86; 0.97)**
multiple times	97	**0.76 (0.62; 0.92)**	**0.81 (0.66; 0.99)**
KARMA cohort (N = 55,044)			
Preeclampsia			
No	2410	1.00 (REF)	1.00 (REF)
Yes	86	**0.77 (0.62; 0.95)**	**0.75 (0.61; 0.93)** ^a^

Model 1 adjusted for calendar period (10-year categories). Model 2 further adjusted for number of births, age at first birth, weight status categories, smoking status and education level. The underlying time scale was attained age. Significant associations are denoted in bold

*Abbreviations*: *IRR* incidence rate ratio, *CI* confidence interval

^a^For model 2 in the KARMA cohort, we additionally adjusted for alcohol use, age at menarche, body shape at age 18 years, physical activity at age 18 years and irregular menstrual cycles in adult life

When investigating a familial aggregated association between preeclampsia and breast cancer, we found a reduced risk of preeclampsia in sisters of patients with breast cancer (OR = 0.89, 95% CI = 0.83; 0.96). This association was confirmed in the genetic analysis. Among women without breast cancer, those who had the highest 10% of PRS for breast cancer were less likely to have preeclampsia during their pregnancy (OR = 0.56, 95% CI = 0.36; 0.86) (Table 3).

**Table 3:** Association between genetic predisposition to breast cancer and preeclampsia in women without breast cancer

			OR (95% CI)	
	Number with non-PE	Number with PE	Model 1	Model 2
Sisters in the nationwide cohort (*N* = 644,483)^a^				
Having sisters with breast cancer				
No	592,547	32,302	1.00 (REF)	1.00 (REF)
Yes	18,785	849	**0.83 (0.77; 0.88)**	**0.89 (0.83; 0.96)**
Genotyped women in the KARMA cohort (*N* = 9263)^b^				
Percentiles of breast cancer polygenic risk score (woman’s own)				
0–40%	3531	175	1.00 (REF)	1.00 (REF)
40–60%	1770	82	0.77 (0.58; 1.03)	0.78 (0.58; 1.04)
60–80%	1743	109	0.78 (0.56; 1.08)	0.78 (0.56; 1.09)
80–90%	866	61	0.77 (0.52; 1.18)	0.77 (0.51; 1.16)
90–100%	881	45	**0.55 (0.36; 0.85)**	**0.56 (0.36; 0.86)**
Standardized continuous			0.92 (0.80; 1.05)	0.92 (0.80; 1.06)

*Abbreviations*: *PE* preeclampsia, *OR* odds ratio, *CI* confidence interval. Significant associations are denoted in bold

^a^Analysis was performed in the Swedish nationwide cohort of pregnant women, and restricted to women with a sister. Model 1 adjusted for number of births. Model 2 further adjusted for age at first birth, weight status categories, smoking status and education level

^b^Analysis was performed among women without breast cancer participating in the KARMA cohort. Model 1 adjusted for number of births and batch effect of genotyping. Model 2 further adjusted for age at first birth, weight status categories, smoking status and education level

**Table 4:** Association between preeclampsia and stratus mammographic density among women in the KARMA cohort (*N* = 43,844)

Condition	Number	Percent density (95% CI)	
		Model 1	Model 2
Preeclampsia (woman herself)			
No	41,583	REF	REF
Yes	2261	-4.72 (-5.45; -3.99)	-2.04 (-2.65; -1.43)
Having a sister with a diagnosis of preeclampsia^a^			
No	3296	REF	REF
Yes	204	-3.51 (-6.12; -0.99)	-2.76 (-4.96; -0.56)

Model 1 adjusted for age at mammography (continuous). Model 2 further adjusted for menopausal status, weight status category, age at menarche, number of births, age at first birth, irregular menstrual cycles, physical activity at age 18 years, education level, smoking status, alcohol consumption, and body shape at age 18 years

^a^We linked the KARMA cohort to the Multi-Generation Register to obtain information on sister relationships among these women, while considering the age of the women in this screening cohort (mostly 40–74 years old). Analysis was restricted to women with a sister in KARMA cohort

### Preeclampsia and mammographic density

In the KARMA cohort for mammographic density analysis, 2261 of the 43,844 women had a previous diagnosis of preeclampsia. These patients with preeclampsia had a lower density (-2.04%, 95% CI = -2.65; -1.43) as compared to the women without preeclampsia, after adjusting for reproductive factors. When restricting the analysis to 3500 women with a sister in the cohort, sisters of patients with preeclampsia also had a reduced percentage density (-2.76%, 95% CI = -4.96; -0.56) compared to women who did not have a sister with preeclampsia (Table 4).

## Discussion

Women diagnosed with preeclampsia had a lower risk of breast cancer as compared to the women without preeclampsia, which is more pronounced in those with multiple occurrences of preeclampsia. Genetic association analysis indicated that sisters of patients with breast cancer and women with a high PRS of breast cancer had a reduced risk of preeclampsia. In addition, patients with preeclampsia and women with sisters with preeclampsia had lower mammographic density.

Few large cohort studies have evaluated the risk of breast cancer in women with preeclampsia. The crude risk estimates (only adjusted for age and calendar period) observed in our nationwide cohort are in agreement with findings from Danish and Norwegian studies [4, 6]. The IRRs in the KARMA cohort were lower than those observed in the nationwide cohort, probably because the KARMA cohort was a screening cohort, with a selection of health-oriented women. However, IRRs in the KARMA cohort are still comparable to those estimates from US populations [11, 25], supporting the generalizability of risk estimates in populations of European ancestry. In contrast, estimates from Chinese, Korean, and Jewish populations showed null or a positive association between preeclampsia and breast cancer [8, 10, 26], suggesting that genetic components might affect this association. The reduced risk of breast cancer in patients with preeclampsia might be confounded by several lifestyle and reproductive factors, including BMI, smoking, education and number of births (see Table 1). However, even after adjusting for these factors (particularly in the analysis of the KARMA cohort, which also included a number of other reproductive factors), the finding of a reduced risk of breast cancer in patients with preeclampsia persisted. A dose response effect of preeclampsia diagnosis observed in the nationwide cohort further supports the association between preeclampsia and breast cancer.

We found an inverse association between preeclampsia and sisters’ history of breast cancer. The effect of family history is confirmed by a previous study using sister controls, where the protective effect of preeclampsia on breast cancer risk was attenuated [7]. Our study also showed an inverse association between preeclampsia and breast cancer genetic risk score, and a further adjustment for breast cancer PRS slightly attenuated the association between breast cancer and preeclampsia, suggesting these genetic components account for part but not all of this inverse association (probably due to power issues and the fact that PRS only accounts for part of the breast cancer genetic information). Both preeclampsia and breast cancer are heritable diseases with heritability of around 30% [27, 28]. A candidate gene approach has discovered about 70 genes to be associated with preeclampsia and some of the genes overlap with the breast cancer susceptibility genes such as *ACE*, *VEGF, IGF1R* and *FLT1* [29–33]. However, results from different studies were inconsistent and no universally acceptable risk gene or SNP for preeclampsia has been defined [34]. For breast cancer genetics, PRS covering a large amount of common genetic variants with small individual effect sizes has already been used for breast cancer risk prediction [35], which is the reason that we used breast cancer PRS to test the association with preeclampsia risk, not vice versa. Overall, our results indicate, probably for the first time, a potential pleiotropic effect of some common genetic factors contributing to the association between preeclampsia and breast cancer.

Our study showed reduced mammographic density in patients with preeclampsia and among patients’ sisters. This finding supports the inverse association between breast cancer and preeclampsia, and the effect of family history. Considering the established association between mammographic density and breast cancer, it is biologically plausible that the association between preeclampsia and breast cancer is to some extent mediated by mammary gland development. Several studies had shown a lower level of insulin-like growth factor (*IGF-1*) in patients with preeclampsia [36], while higher *IGF-1* is found in patients with breast cancer and women with high mammographic density [37, 38]. A lower level of free vascular endothelial growth factor (*VEGF*) was also observed among patients with preeclampsia [39], which is a key component in breast tumor angiogenesis [40] and mammary gland development [41]. In addition, *VEGF* and *IGF-1* receptor genetic variations may modify the inverse association between gestational hypertension (a symptom of preeclampsia) and mammographic density [42], and *IGF1R* genetic variations may predict breast cancer risk in patients with preeclampsia [33], further supporting the role of genetic factors in the association between preeclampsia and breast cancer, and suggesting future studies on these potential genetic factors are needed. The exact mechanisms responsible for the inverse association between preeclampsia and breast cancer could therefore be used to evaluate women’s risk of breast cancer.

The main strength of our study is the use of both nationwide registers and self-reported data to identify a reduced risk of breast cancer in patients with preeclampsia and the effect of inherited factors. Our mammographic density findings further supported the association between preeclampsia and breast cancer.

We acknowledge several limitations of this study. Although a diagnosis of preeclampsia in the Swedish Medical Birth Register has an approximately 93% validation [43], self-reported data on preeclampsia in the KARMA cohort have not been validated and may be limited by recall bias. In the KARMA cohort, we selected women with at least one child and who were alive until 2011. While excluding women who died before 2011 may have introduced survival bias, meaning the KARMA cohort may represent a healthier population, we speculate that this would only attenuate the protective effect of preeclampsia and not influence our conclusions. In addition, we actually found a higher incidence rate of breast cancer in the KARMA cohort than the nationwide cohort, suggesting a selection of health-oriented women with a higher level of education or a family history of breast cancer in this screening cohort [16]. Third, we cannot rule out the possibility that the observed association between breast cancer PRS and preeclampsia may be due to chance, since we can only observe a significant risk reduction of preeclampsia with the top 10% of PRS (because of the relatively small sample size and the weak association). However, evidence from the nationwide cohort supports an inverse association between a genetic predisposition to breast cancer and preeclampsia, and there is a significant trend of greater preeclampsia risk reduction in women with higher PRS (*p* for trend = 0.01).

## Conclusion

We found that women with previous preeclampsia had a lower risk of breast cancer and lower mammographic density than women without a diagnosis of preeclampsia. This finding could partly be explained by genetic factors, shared between breast cancer and preeclampsia. The exact mechanism underlying genetic association between these two diseases remains to be defined. In addition, our results suggest that history of preeclampsia should be considered in the evaluation of women’s risk of breast cancer.

## Additional file

Additional file 1: Table S1. List of single nucleotide polymorphisms (SNPs) used for constructing the polygenic risk score (PRS) for breast cancer. **Figure S1.** Stunkard Figure Rating Scale illustrating body sizes ranging from extreme thinness (category 1) to obesity (category 9). (DOCX 59 kb)

## Abbreviations

BMI: Body mass index
CI: Confidence interval
ER+: Estrogen receptor positive
ER-: Estrogen receptor negative
GWAS: Genome-wide association studies
ICD: International Classification of Diseases
IRR: Incidence rate ratio
KARMA: Karolinska Mammography Project for Risk Prediction of Breast Cancer
MLO: Mediolateral oblique
OR: Odds ratio
PRS: Polygenic risk score
SNPs: Single nucleotide polymorphisms
