# Supplementary material for: Inherited factors contribute to an inverse association between preeclampsia and breast cancer
Source: Breast Cancer Res. 2018 Jan 23;20:6. doi: 10.1186/s13058-017-0930-6 (PMC5782395; doi:10.1186/s13058-017-0930-6)

**Additional file 1**

**Table S1**. List of single nucleotide polymorphisms (SNPs) used for constructing the polygenic risk score (PRS) for breast cancer.

| SNP NAME | EFFECT SIZE | SNP NAME | EFFECT SIZE | SNP NAME | EFFECT SIZE | SNP NAME | EFFECT SIZE |
| --- | --- | --- | --- | --- | --- | --- | --- |
| rs116095464 | 0.0592 | rs117618124 | -0.12186 | rs6562760 | 0.04724 | rs11249433 | 0.10649 |
| rs6597981 | 0.04502 | rs12493607 | 0.04756 | rs6472903 | 0.0618 | rs11199914 | -0.04115 |
| rs10069690 | 0.05673 | rs2012709 | 0.02069 | rs2943559 | 0.09666 | rs2981578 | -0.20635 |
| rs3215401 | -0.07005 | rs2284378 | 0.00256 | rs745570 | -0.02764 | rs35054928 | -0.24233 |
| rs11242675 | -0.00038 | rs11571833 | 0.29858 | rs13329835 | 0.06927 | rs45631563 | -0.20712 |
| rs3817198 | 0.04781 | rs13365225 | -0.09442 | rs704010 | -0.07148 | rs58847541 | 0.07327 |
| rs6762644 | 0.05255 | rs2236007 | -0.06871 | rs12207986 | 0.0336 | rs77528541 | -0.05627 |
| rs2380205 | -0.02293 | rs738321 | -0.05154 | rs7707921 | 0.04021 | rs13281615 | 0.10035 |
| rs16991615 | 0.09571 | rs6815814 | 0.05672 | rs17529111 | 0.02194 | rs11780156 | 0.04974 |
| rs67958007 | 0.08237 | chr22:39359355 | 0.09697 | 4:84370124 | -0.03488 | rs10760444 | -0.03297 |
| rs113577745 | 0.07548 | rs72826962 | 0.18359 | rs202049448 | -0.05204 | rs11820646 | 0.03888 |
| rs9348512 | 0.00445 | rs6001930 | 0.1174 | rs13066793 | -0.06161 | rs6569648 | 0.05848 |
| rs616488 | -0.05785 | rs4233486 | 0.03493 | rs4496150 | -0.0433 | rs4593472 | -0.03184 |
| rs78269692 | 0.08354 | rs73161324 | 0.054 | rs17426269 | 0.04726 | rs6596100 | -0.05744 |
| rs204247 | -0.03624 | rs79724016 | -0.07592 | rs10022462 | 0.04011 | rs8176636 | 0.03291 |
| rs2594714 | -0.02959 | rs6507583 | -0.08015 | rs10474352 | -0.05862 | rs11977670 | 0.06096 |
| rs12422552 | 0.05611 | rs2532263 | -0.05417 | rs2290203 | -0.05839 | rs34207738 | 0.05547 |
| rs13162653 | -0.01211 | rs3760982 | -0.05049 | rs6964587 | 0.03395 | rs720475 | -0.03749 |
| rs3819405 | -0.04546 | rs10941679 | 0.14228 | rs941764 | 0.03356 | rs12405132 | -0.03075 |
| rs2823093 | -0.06228 | rs71338792 | 0.04903 | rs11627032 | -0.03915 | rs9485372 | -0.04584 |
| rs67397200 | 0.02821 | rs28512361 | 0.04968 | rs17268829 | 0.04834 | rs12048493 | 0.04183 |
| rs4808801 | -0.07028 | rs1707302 | 0.03643 | rs140936696 | -0.04083 | rs3757322 | 0.07948 |
| rs2992756 | -0.06077 | rs6796502 | -0.0844 | rs17356907 | -0.0906 | rs9397437 | 0.16059 |
| rs12710696 | -0.02531 | rs6122906 | 0.04874 | rs9833888 | 0.05556 | rs2747652 | 0.06131 |
| rs2965183 | 0.04159 | rs72749841 | -0.06783 | rs71559437 | -0.06785 | rs4971059 | 0.05186 |
| rs2223621 | -0.03649 | rs35951924 | -0.05293 | rs514192 | -0.04424 | rs1432679 | -0.07649 |
| rs7971 | -0.04136 | rs140850326 | -0.0324 | rs10623258 | 0.0381 | rs4562056 | 0.0481 |
| rs7072776 | -0.05064 | rs4784227 | 0.2044 | rs9790517 | 0.03486 | rs58058861 | 0.06186 |
| rs1011970 | 0.06341 | rs2787486 | -0.0762 | rs12546444 | -0.06933 | rs2016394 | -0.04629 |
| rs11814448 | 0.11579 | rs17817449 | -0.0537 | rs10759243 | 0.0614 | rs1550623 | 0.04915 |
| rs527616 | 0.03382 | rs11075995 | -0.03376 | rs10816625 | 0.10113 | rs6828523 | -0.09718 |
| rs1436904 | -0.04891 | rs28539243 | 0.04943 | rs13294895 | 0.05597 | rs35383942 | 0.11704 |
| rs6725517 | -0.04105 | rs62355902 | 0.16344 | rs676256 | 0.08906 | rs1830298 | -0.0561 |
| rs71557345 | -0.08273 | rs2432539 | -0.03342 | rs6882649 | 0.02881 | rs6678914 | 0.00317 |
| rs4973768 | 0.10012 | rs10472076 | 0.02513 | rs71801447 | 0.0831 | rs4951011 | 0.04219 |
| rs7297051 | -0.11548 | rs1353747 | -0.0413 | rs11552449 | 0.0367 | rs4245739 | -0.02282 |
| rs17156577 | 0.04968 | rs1053338 | 0.04514 | rs7904519 | 0.03004 | rs11117758 | -0.0548 |
| rs9257408 | 0.01764 | rs10995201 | -0.10601 | rs1292011 | -0.08245 | rs4442975 | -0.11867 |
| rs4577244 | 0.01269 | rs3903072 | -0.02988 | rs13267382 | -0.03129 | rs34005590 | -0.19504 |
| rs17879961 | 0.22752 | rs2588809 | -0.05622 | rs7529522 | 0.06048 | rs16857609 | 0.06176 |
| rs146699004 | -0.03277 | rs999737 | -0.09552 | rs1895062 | -0.06633 | rs12479355 | -0.03876 |
| rs9693444 | -0.06128 | rs75915166 | 0.24973 | rs206966 | 0.04464 | rs72755295 | 0.13592 |
| rs132390 | -0.04099 | rs6805189 | -0.03247 | rs4849887 | 0.09619 |  |  |

**Figure S1** Stunkard Figure Rating Scale illustrating body sizes ranging from extreme thinness (category 1) to obesity (category 9)


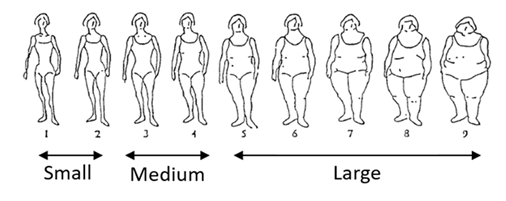

Supplement: Additional file 1: Table S1. — List of single nucleotide polymorphisms (SNPs) used for constructing the polygenic risk score (PRS) for breast cancer. Figure S1. Stunkard Figure Rating Scale illustrating body sizes ranging from extreme thinness (category 1) to obesity (category 9). (DOCX 59 kb) [file 13058_2017_930_MOESM1_ESM.docx]
